# Flow-dependent shear stress affects the biological properties of pericyte-like cells isolated from human dental pulp

**DOI:** 10.1186/s13287-023-03254-2

**Published:** 2023-02-18

**Authors:** Giulia Bertani, Rosanna Di Tinco, Laura Bertoni, Giulia Orlandi, Alessandra Pisciotta, Roberto Rosa, Luca Rigamonti, Michele Signore, Jessika Bertacchini, Paola Sena, Sara De Biasi, Erica Villa, Gianluca Carnevale

**Affiliations:** 1 Department of Surgery, Medicine Dentistry and Morphological Sciences With Interest in Transplant, University of Modena and Reggio Emilia, Modena, Italy; 2 Department of Engineering Sciences and Methods, University of Modena and Reggio Emilia, Modena, Italy; 3 Department of Chemical and Geological Sciences, University of Modena and Reggio Emilia, Modena, Italy; 4 RPPA Unit, Proteomics Area, Core Facilities, Istituto Superiore di Sanità, Rome, Italy; 5 Department of Medical and Surgical Sciences for Children and Adults, University of Modena and Reggio Emilia, Modena, Italy

**Keywords:** Pericytes, Neural crest, Dental pulp stem cells, Flow-dependent shear stress, Angiogenesis, Inflammation

## Abstract

**Background:**

Human dental pulp stem cells represent a mesenchymal stem cell niche localized in the perivascular area of dental pulp and are characterized by low immunogenicity and immunomodulatory/anti-inflammatory properties. Pericytes, mural cells surrounding the endothelium of small vessels, regulate numerous functions including vessel growth, stabilization and permeability. It is well established that pericytes have a tight cross talk with endothelial cells in neoangiogenesis and vessel stabilization, which are regulated by different factors, i.e., microenvironment and flow-dependent shear stress. The aim of this study was to evaluate the effects of a pulsatile unidirectional flow in the presence or not of an inflammatory microenvironment on the biological properties of pericyte-like cells isolated from human dental pulp (hDPSCs).

**Methods:**

Human DPSCs were cultured under both static and dynamic conditions with or without pre-activated peripheral blood mononuclear cells (PBMCs). Pulsatile unidirectional flow shear stress was generated by using a specific peristaltic pump. The angiogenic potential and inflammatory properties of hDPSCs were evaluated through reverse phase protein microarrays (RPPA), confocal immunofluorescence and western blot analyses.

**Results:**

Our data showed that hDPSCs expressed the typical endothelial markers, which were up-regulated after endothelial induction, and were able to form tube-like structures. RPPA analyses revealed that these properties were modulated when a pulsatile unidirectional flow shear stress was applied to hDPSCs. Stem cells also revealed a downregulation of the immune-modulatory molecule PD-L1, in parallel with an up-regulation of the pro-inflammatory molecule NF-kB. Immune-modulatory properties of hDPSCs were also reduced after culture under flow-dependent shear stress and exposure to an inflammatory microenvironment. This evidence was strengthened by the detection of up-regulated levels of expression of pro-inflammatory cytokines in PBMCs.

**Conclusions:**

In conclusion, the application of a pulsatile unidirectional flow shear stress induced a modulation of immunomodulatory/inflammatory properties of dental pulp pericyte-like cells.

**Supplementary Information:**

The online version contains supplementary material available at 10.1186/s13287-023-03254-2.

## Introduction

Pericytes, vascular mural cells, are embedded in the basement membrane of blood microvessels including capillaries, precapillary arterioles and post-capillary venules.

Their role consists in reinforcing vascular structure and regulating microvascular blood flow, although they do not only serve as scaffolding as conventionally thought, but communicate with endothelial cells by direct physical contact and paracrine signaling pathways [1]. Besides exerting a primary function in endothelial stabilization, they indeed contribute to angiogenesis by differentiating into endothelial cells and forming new tubes [2]. It is noteworthy that the biological behavior of pericytes relies on the control exerted by endothelial cells (ECs) through the activation of multiple pathways and the release of pro-angiogenic factors, such as platelet-derived growth factor B (PDGF-B) [3] and angiopoietin 1 (Ang-1)/angiopoietin 2 (Ang-2)/Tie2, which are critically involved in embryonic or pathological angiogenesis. Moreover, it is known that interactions between ECs and pericytes are affected not only by biochemical stimuli but also by mechanical/physical factors, including blood pressure and flow-dependent shear stress, which may influence their cross talk and cell functions as well [4, 5].

Indeed, flow-dependent shear stress (FSS) plays a pivotal role in different pathological conditions, including chronic liver diseases. FSS alterations might affect the interstitial microenvironment and resident cells biological behavior, leading to the inflammatory process and a pro-fibrotic transition. These changes may support each other. For instance, in hepatocellular carcinoma the evolution of fibrosis associated with unresolved hepatic inflammatory process and the inherent progressive alteration of the normal laminar flow into a much more turbulent flow could be a key event in starting the process of endothelial activation [6].

In order to better clarify the role of pericytes in FSS-induced pathological conditions, our study used a pericyte-like cell population isolated from human dental pulp. Human dental pulp pericyte-like cells are entrapped in the loose connective tissue and are located in the perivascular area within dental pulp and express the typical pericyte markers nestin, PDGFRβ, smooth muscle actin α (αSMA) and VEGF [7].

In light of their embryological origin, hDPSCs own peculiar stemness properties that confer them high proliferative rate, immunomodulatory properties and low immunogenicity [8] as well as the ability to achieve the commitment into different cell lineages in vitro [9–13]. hDPSCs have also been shown to support tissue regeneration/homeostasis in vivo, by exerting direct and/or paracrine mechanisms on host progenitor cells [9, 11, 12]. It has been demonstrated that hDPSCs co-cultured with pre-activated peripheral blood mononuclear cells (PBMCs) exert immunomodulatory/anti-inflammatory properties by activating immune-checkpoint, including PD1/PD-L1 and Fas/FasL pathways, and by modulating the expression of the cytokines mainly involved in inflammatory response [8, 13, 14].

As established in the literature, stem cell properties are regulated by intrinsic mechanisms and extrinsic cues that emanate from the surrounding microenvironment. Inflammation is one of them [15]. To this regard, understanding the cross talk between inflammation and stem cells has aroused a huge interest among the scientific community, since it might elucidate the mechanisms activated by stem cells to respond to tissue damage and how to shape them to preserve the tissue homeostasis.

Based on these premises, to better understand the role of pericyte-like cells isolated from dental pulp, our study aimed to evaluate how flow-dependent shear stress may affect the biological properties of pericyte-like cells isolated from human dental pulp (hDPSCs) to predict their potential role in physiological and pathophysiological conditions.

## Methods

### Human dental pulp stem cells (hDPSCs) isolation and culture

This study was carried out in compliance with the recommendations of Comitato Etico Provinciale–Azienda Ospedaliero-Universitaria di Modena (Modena, Italy), which provided the approval of the protocol [ref. number 3299/CE; 5 September 2017]. Dental pulp tissue was obtained, after routine dental extraction, from third molars of adult subjects (*n* = 3; 30–35 years) who gave their written informed consent according to the Declaration of Helsinki. Cell isolation from dental pulp was carried out as previously described [9].

Briefly, the dental pulp was harvested from the teeth and enzymatically digested by means of 3 mg/mL type I collagenase and 4 mg/mL dispase in α-MEM. After filtering with a 100 μm cell strainer, the cell suspension was resuspended in α-MEM supplemented with 10% heat-inactivated fetal bovine serum (FBS), 2 mM L-glutamine, 100 U/mL penicillin, 100 μg/mL streptomycin at 37 °C and 5% CO_2_. Following cell expansion, hDPSCs were immune-selected by magnetic activated cell sorting (MACS), using MACS® separation kit according to manufacturer’s instructions. Mouse IgM anti-STRO-1 and rabbit IgG anti-c-Kit primary antibodies (Santa Cruz, Dallas, TX, USA) were used and revealed by the following magnetically labeled secondary antibodies: anti-mouse IgM and anti-rabbit IgG (Miltenyi Biotec, Bergisch Gladbach, Germany).

### Endothelial differentiation of hDPSCs

STRO-1^+^/c-Kit^+^ hDPSCs were induced toward the endothelial differentiation according to established protocols present in the literature [16]. Briefly, hDPSCs were cultured for 3, 5 and 7 days, respectively, in cell culture dishes at a density of 3 × 10^3^ cells/dishes in endothelial medium consisting in EGM-2 supplemented with 2% FBS, 0.4% hFGF-B, 0.04% hydrocortisone, 0.1% VEGF, 0.1% R3-IGF-1, 0.1% ascorbic acid, 0.1% hEGF, 0.1% GA-1000, 0.1% heparin (EGM-2 Endothelial Medium BulletKit, Lonza Group Ltd, Basel, Switzerland). In order to evaluate the ability of hDPSCs to form tubule-like structure, tube formation analysis was performed using Matrigel® Matrix (Corning, New York, NY, USA) solution according to manufacturers’ instructions. Undifferentiated hDPSCs were used as controls. The endothelial tubule-like vascular network formation was observed after 5 h of incubation under an inverted microscope. Images were acquired with a Nikon inverted microscope. Tube-like structures were counted per 15,000 μm^2^ areas on 5 different fields from 5 different experiments.

### Human umbilical vein endothelial cells (HUVECs) culture

HUVECs (ATCC, USA) were incubated in Vascular Cell Basal Medium (ATCC, USA) supplemented with Endothelial Cell Growth Kit-VEGF (ATCC, USA) at 37 °C, 5% CO_2_ humidified incubator. Culture medium was changed every 2–3 days. Cells were used as controls.

### Human dental pulp stem cells cultured under dynamic conditions

**Fig. 1:**
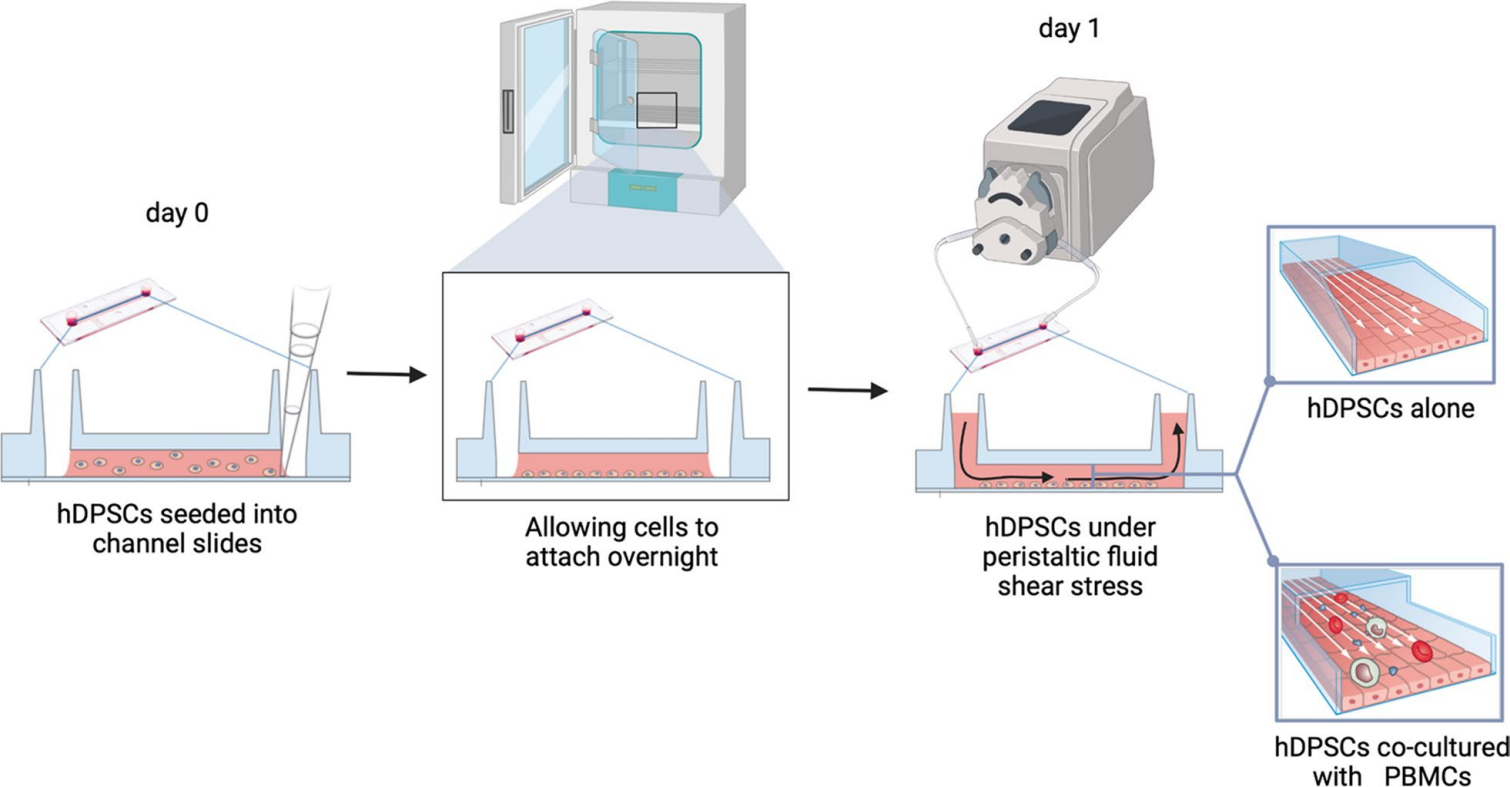
Experimental workflow of hDPSCs cultured under dynamic conditions. Illustrations were edited from Ibidi

Immune-selected hDPSCs suspension was filled into the channel slide of Ibidi μ-slides I Luer (height 0.4 mm) chambers (Ibidi, Gräfelfing, Germany) and allowed to attach overnight in standard culture medium at 37 °C, 5% CO_2_ humidified incubator. The experimental setup was carried out according to the manufacturer’s instructions (Ibidi) and is summarized in Fig. 1. Briefly, after connecting the channel slide to the peristaltic pump (React 4 Life, Peristaltic Pump R100-1 J), a unidirectional pulsatile flow of 1.5 dyn/cm^2^ was applied with a 90 min time span after minor changes to the protocols previously described [17, 18].

### Peripheral blood mononuclear cells (PBMCs) isolation

PBMCs were isolated from fresh venous blood taken from 5 healthy adult volunteers. PBMCs were isolated by using Histopaque (Sigma-Aldrich), according to the manufacturer’ instructions and pre-activated as previously described [14]. In particular, direct co-culture system was established by seeding hDPSCs/PBMCs at a cell ratio 1:10 and cultured in RPMI 1640 medium supplemented with 10% FBS, 2 mM glutamine, 100 units/mL penicillin and 100 mg/mL streptomycin. Direct co-culture was performed both in static and dynamic conditions, respectively.

### Immunofluorescence and immunohistochemistry analyses

*Immunofluorescence.* Cells were fixed with 4% paraformaldehyde at 4 °C for 15 min and then permeabilized with 0.3% Triton X-100 for 5 min. After blocking with 3% bovine serum albumin (BSA) in pH 7.4 phosphate buffer saline (PBS) for 1 h, the cells were incubated at 4 °C overnight with the following primary antibodies: rabbit anti-PDGFR-β (Cell Signaling Technology, Trask Lane Danvers, MA, USA), rabbit anti-NG2 (Abcam), mouse anti-α smooth muscle actin (αSMA; Invitrogen), mouse anti-VEGF (Invitrogen, Waltham, MA, USA), rabbit anti-eNOS (Merck Millipore, Burlington, MA, USA), rabbit anti-ANGPT1 (GeneTex, California, USA), rabbit anti-Tie2 (Abcam, Cambridge, UK), mouse anti-STRO-1, rabbit anti-c-Kit (all diluted 1:100) Cell Signaling Technology, Trask Lane Danvers, MA, USA). Subsequently, the cells were incubated at 37 °C for 1 h at room temperature with the following secondary antibodies: goat anti-mouse Alexa488, goat anti-rabbit Alexa 546, goat anti-mouse Alexa546, goat anti-rabbit Alexa488 (all diluted 1:200; Thermo Fisher). Cell morphology was evaluated through immunolabeling with TRITC-conjugated, anti-phalloidin antibody (Abcam, Cambridge, UK).

Nuclei were counterstained with 1 μM 4,6-diamidino-2-phenylindole (DAPI) (Sigma-Aldrich). Images were captured by using Nikon A1 confocal laser scanning microscope as previously described [8].

*Immunohistochemistry.* Dental pulp samples were fixed in 4% paraformaldehyde in PBS and then paraffin-embedded. Five-micrometer-thick sections were cut, and immunohistochemistry carried out as previously described [9].

The following primary antibodies were used: mouse anti-VEGF (Invitrogen, Waltham, MA, USA) and rabbit anti-PDGFR-β (1:100, Cell Signaling Technology, Trask Lane Danvers, MA, USA). The sections were lightly counterstained with hematoxylin, mounted, and analyzed under an optical Nikon microscope.

### FACS analysis

In order to evaluate the expression of the typical mesenchymal stem cells (MSCs) markers, immune-selected hDPSCs, both in static and dynamic culture conditions, underwent FACS analysis against CD73, CD90, CD105, CD34, CD45, HLA-DR, as formerly described by Conserva et al. [19]. Following trypsin dissociation, cells were resuspended in culture medium and were stained with the following fluorochrome-conjugated antibodies (Abs): anti-human-CD73-PE-CY7, anti-human-CD90-FITC, anti-human-CD105-APC, anti-human-CD45-PE, and anti-human-HLADR-PE-CY7 (all from BD Biosciences, Franklin Lakes, NJ, USA); and anti-human-CD34-ECD (Beckman Coulter, Fullerton, CA, USA). A minimum of 10,000 cells per sample was acquired and analyzed by using the Attune Acoustic Focusing Flow Cytometer (Attune NxT, Thermo Fisher, Waltham, MA, USA). Data were analyzed by FlowJo 9.5.7 (Treestar, Inc., Ashland, OR, USA) under MacOS 10.

### Reverse phase protein microarray

For RPPA analysis, protein extracts were processed with a RIPA-like lysis buffer (LB) composed of TPER (Thermo Fisher Scientific, Waltham, MA, USA) and additioned with 300 mM NaCl and protease and phosphatase inhibitors cocktails (Merck Millipore, Burlington, MA, USA). Cell pellets were resuspended in an adequate volume of ice-cold LB, incubated on ice for 30 min and spun for 15 min at 12,000 rpm on a refrigerated centrifuge as previously described [20–22]. The protocols we used for RPPA analysis were previously optimized [20, 21]. Protein extracts were prepared by diluting 20 μg of protein extract with Novex™ Tris–Glycine SDS Sample Buffer 2X (Thermo Fisher Scientific, Waltham, MA, USA) as per the recipe by Laemmli [23]. Subsequently, RPPA samples were loaded onto an Aushon 2470 contact pin arrayer (Quanterix, MA, USA) and printed in technical triplicates onto nitrocellulose-coated slides (Grace Bio-Labs, OR, USA). The number of slides printed has been designed to include, but not limit the staining to at least 40 antibodies. In detail, a fraction of the printed slides was reserved for fluorescent detection of total protein content via Sypro Ruby (Thermo Fisher Scientific, Waltham, MA, USA) and the remaining slides were stored at − 20 °C for subsequent antibody staining. Immediately prior to staining, slides were treated with 1 × ReBlot Mild Solution (Merck Millipore, Burlington, MA, USA) for 15 min, washed 2 × 5 min with PBS (Euroclone, Milan, Italy) and incubated for 2 h in a blocking solution containing 2% I-Block powder (Thermo Fisher Scientific, Waltham, MA, USA) and 0.1% Tween 20 in PBS. Immunostaining was carried out using a commercially available signal amplification kit (Agilent, CA, USA) based on catalyzed reporter deposition (CARD) [24]. Primary antibody binding was detected via secondary staining with biotinylated goat anti-rabbit IgG H + L (1:7500) (Vector Laboratories, CA, USA) or rabbit anti-mouse IgG (1:5000) (Agilent, CA, USA), followed by signal amplification and a tertiary streptavidin conjugated with IRDye680LT fluorophore (LI-COR Biosciences, NE, USA). Negative control slides were reserved on a per-staining run basis and incubated with secondary antibodies alone. Primary antibodies against the selected (phospho-)targets are part of a larger collection of antibodies that have undergone in-house validation for RPPA analysis using western blotting [25]. MicroVigene v5.2 (VigeneTech, Carlisle, MA) software was used for spot detection, local background subtraction, replicate averaging as well as for background amplification signal subtraction and total protein content normalization. Throughout the manuscript and in the figures, the above-described normalized RPPA data are referred to as normalized RPPA intensity or levels and are expressed in arbitrary units (A.U.). Raw RPPA data are reported in Additional file 1: Table S1.

### Western blot analysis

Where indicated, cell pellets were analyzed in western blot (WB). Whole cell lysates were obtained as formerly reported [10]. Briefly, 40 μg of protein extract per specimen was quantified by a Bradford Protein Assay (Bio-Rad), and then, SDS–polyacrylamide gel electrophoresis and subsequent protein transfer to nitrocellulose membranes were performed. The following antibodies were used: rabbit anti-PDGFR-β (Cell Signaling Technology, Trask Lane Danvers, MA, USA), rabbit anti-Tie2 (Abcam, Cambridge, UK), rabbit anti-eNOS (Merck Millipore, Burlington, MA, USA), rabbit anti-ANGPT1 (GeneTex California, USA), mouse anti-VEGF (Invitrogen, Waltham, MA, USA) and rabbit anti-cleaved caspase 3 (Cell Signaling Technology) diluted 1:1,000 in Tris-buffered saline (TBS) Tween 20 0.1%, plus 2% BSA and 3% non-fat milk and incubated overnight at 4 °C. Membranes were then incubated for 1 h at room temperature with HRP-conjugated anti-mouse and anti-rabbit secondary antibodies, diluted 1:2,000 in TBS Tween 20 0.1% plus 2% BSA and 3% non-fat milk. Membranes were then visualized by using Clarity Western ECL Substrate (Bio-Rad, Alfred Nobel Drive Hercules, CA, USA), according to the manufacturer’s instructions. Anti-actin antibody was used as control of protein loading. Densitometry of PDGFR-β, Tie2, eNOS, ANGPT1, VEGF and cleaved caspase 3 was carried out with Fiji ImageJ software. An equal area was selected inside each band, and the mean of gray levels (in a 0–256 scale) was calculated. Data were then normalized to values of background and of control actin band [10].

### RNA purification and quantitative real-time PCR

Total RNA extraction was performed by using the PureLink™ RNA Micro Kit (Invitrogen, Waltham, MA, USA) according to manufacturer’s instructions. RNA integrity and quantification were analyzed by a spectrophotometric method by using a NanoDrop 2000 device (Thermo Fisher Scientific, Waltham, MA, USA). Total RNA (1 μg) was reverse transcribed to cDNA using the QuantiTect Reverse Transcription Kit (Qiagen, Hilden, Germany), according to manufacturer’s instructions. Levels of mRNA were quantitatively determined on a QuantStudio™ 3 Real-Time PCR System (Applied Biosystems, Thermo Fisher Scientific, Waltham, MA, USA) using the QuantiFast SYBR Green PCR Kit according to the manufacturer’s instructions (Qiagen, Hilden, Germany). PCR primer sequences were as follows: hIL-2 (F: AAAGAAAACACAGCTACAACTGG, R: GAAGATGTTTCAGTTCTGTGGC); hIFNγ (F: GCATCGTTTTGGGTTCTCTTG R: AGTTCCATTATCCGCTACATCTG), hTNFα (F: ACTTTGGAGTGATCGGCC, R: GCTTGAGGGTTTGCTACAAC), hIL-6 (F: CCACTCACCTCTTCAGAACG, R: CATCTTTGGAAGGTTCAGGTTG), hIL-10 (F: CAGAGTGAAGACTTTCTTTCAAATG, R: CCTTTAACAACAAGTTGTCCAGC); hRPLP0 (F: TACACCTTCCCACTTGCTGA, R: CCATATCCTCGTCCGACTCC).

The relative gene expression quantification was performed using the comparative threshold (Ct) method (ΔΔCt), where relative gene expression level equals 2^−ΔΔCt^. The obtained fold changes in gene expression were normalized to the housekeeping gene RPLP0.

The real-time PCR data were processed by one-way ANOVA followed by Newman–Keuls post hoc test (GraphPad Prism Software version 5 Inc., San Diego, CA, USA). The data were expressed as the mean value ± standard deviation from three independent experiments. For all tested groups, the statistical significance was set up at *P* < 0.05.

### Statistical analysis

All the experiments were performed in triplicate. Data were expressed as mean ± SD. Differences between two experimental conditions were analyzed by unpaired Student’s t test. Differences among three or more experimental samples were analyzed by ANOVA followed by Newman–Keuls post hoc test (GraphPad Prism Software version 5 Inc., San Diego, CA, USA). Significance was set at *P* < 0.05.

## Results

### Pericytes-like features of hDPSCs

**Fig. 2:**
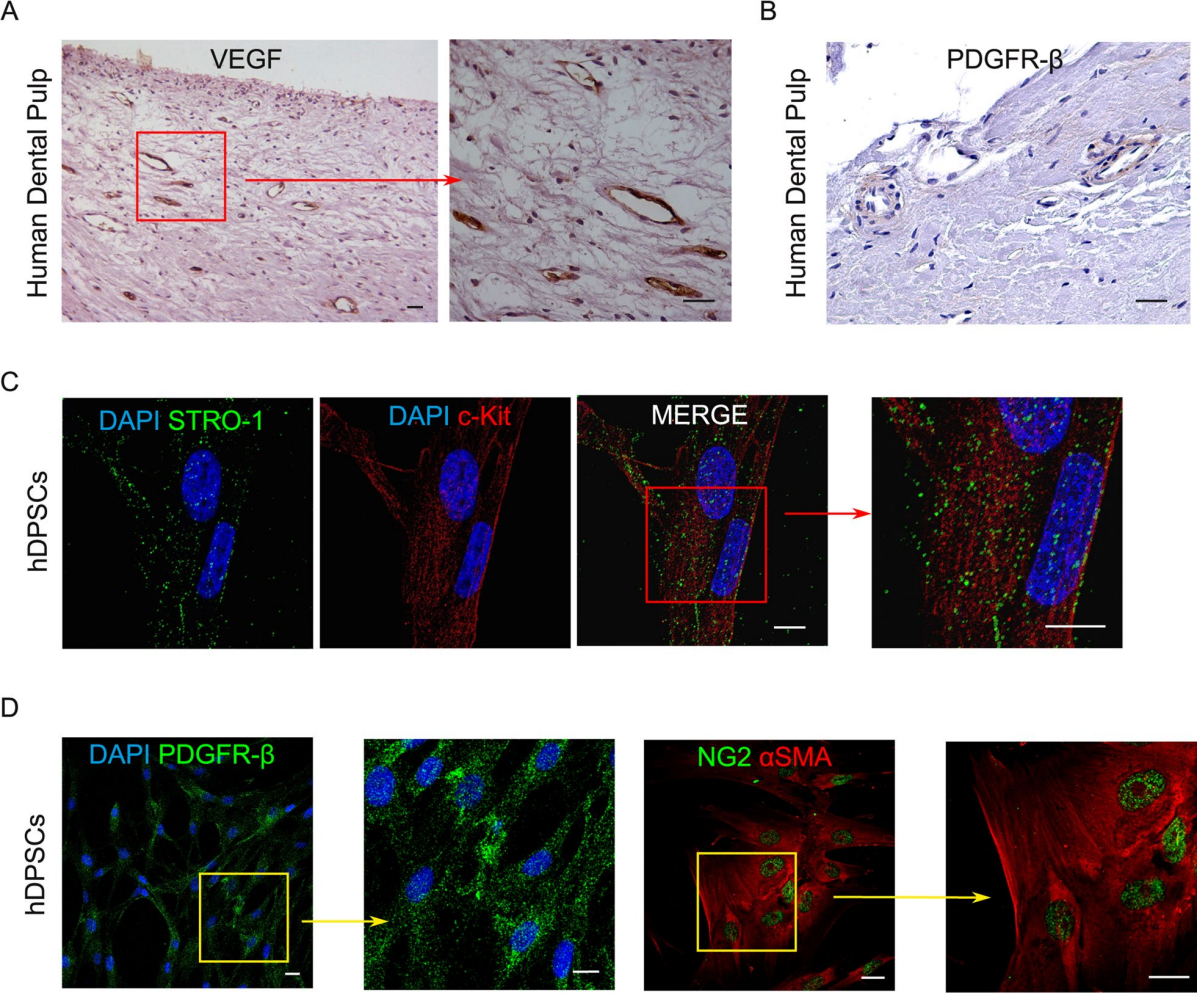
Immune-phenotypical characterization of hDPSCs. Immunohistochemistry of VEGF expression **A** and PDGFR-β **B** in human dental pulp tissue. Nuclei were counterstained with hematoxylin. Red square in A indicates higher magnification on the right. Scale bar: 50 μm. **C** Immunofluorescence analysis of STRO-1 and c-Kit in immune-selected hDPSCs. Red square indicates higher magnification highlighting the expression pattern of both markers. Scale bar: 10 μm. **D** Immunofluorescence analysis performed on STRO-1 + /c-Kit + hDPSCs shows the expression of PDGFR-β, NG2 and αSMA. Nuclei were stained with DAPI (blue). Yellow squares indicate higher magnifications of the related fields. Scale bar: 10 μm

Human dental pulp is a soft connective tissue entrapped within the pulp chamber of the tooth and is considered an interesting source of adult stem cells, i.e., hDPSCs. As shown in Fig. 2A, dental pulp tissue is largely vascularized as remarked by the immunohistochemical labeling against VEGF. Moreover, cells surrounding blood vessels express PDGFR-β, a typical pericyte marker (Fig. 2B). After dental pulp digestion, hDPSCs were isolated and expanded in vitro*.* Subsequently, through magnetic cell sorting, a pure stem cell population expressing STRO-1 and c-Kit was obtained and confirmed by immunofluorescence analysis (Fig. 2C). As reported in Fig. 1D, the immune-selected STRO-1 + /c-Kit + hDPSCs population expressed PDGFR-β, NG2 and αSMA, confirming the pericyte-like cells phenotype (Fig. 2D).

### Angiogenic potential of pericytes-like cells

**Fig. 3:**
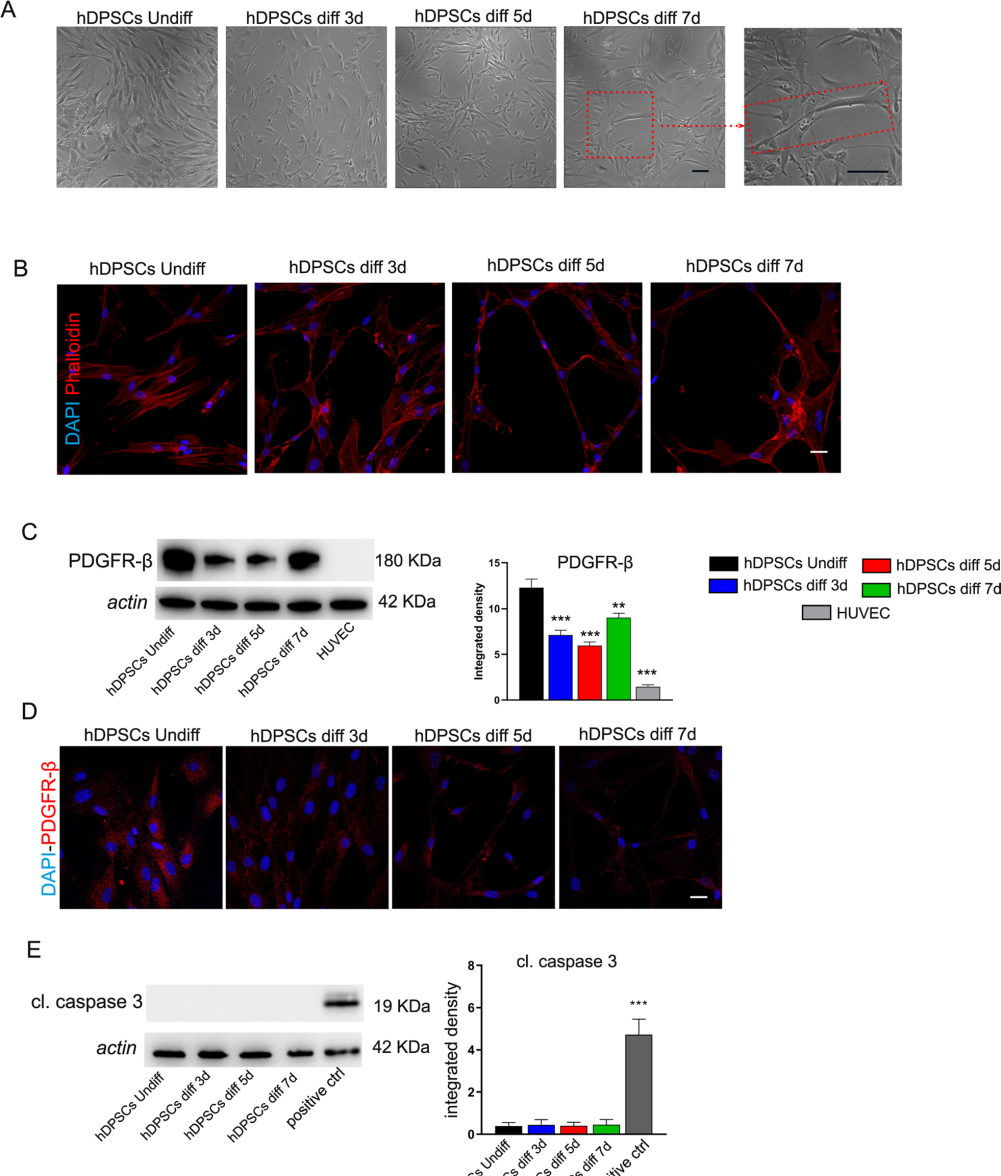
Endothelial differentiation of hDPSCs.** A** Morphological analysis of undifferentiated and endothelial differentiated hDPSCs after 3, 5 and 7 days of induction. Red dashed square and rectangle highlight the cord-like vessel morphology of differentiated hDPSCs. Scale bar: 50 μm. **B** Phalloidin staining shows the morphological rearrangement of hDPSCs toward endothelial lineage. Scale bar: 10 μm. **C–D** Western blot and immunofluorescence analyses of PDGFR-β in hDPSCs after endothelial differentiation. hDPSCs undiff and HUVEC were used as controls. **E** Western blot analysis of cleaved caspase 3 in hDPSCs following endothelial differentiation. Full-length blots are presented in Additional file 1: Figure S1A. Densitometry analysis is shown in histograms. Data are represented as mean ± SD, and statistical analysis on C and E was performed by one-way ANOVA followed by Dunnett post hoc test; ***P < 0.001, **P < 0.01 vs hDPSCs undiff (*n* = 3) (**C)**, ***P < 0.001 positive ctrl vs hDPSCs (*n* = 3) (**E**)

Pericytes have been strongly associated with vessel formation and are involved in hemodynamic processes [26]. They are able to adhere to tissue culture plastic and to differentiate in vitro into different cell lineages [27]. The first aim of our study was to confirm the angiogenic abilities of hDPSCs. After 3, 5 and 7 days of induction toward endothelial differentiation, the commitment of hDPSCs was investigated through morphological, Western Blot and immunofluorescence analyses. Overall, during the induction of the endothelial differentiation, hDPSCs lost their typical fibroblast-like morphology. Morphological changes in elongated endothelial cells were appreciable after 3 and 5 days of commitment. After 7 days of induction, cells assembled into a linear cord-like vessel (Fig. 3A, red dashed rectangle, higher magnification). These data were further supported by phalloidin staining as shown in Fig. 3B. In parallel, WB analysis of differentiated hDPSCs revealed a statistically significant decrease in PDGFR-β expression when compared to undifferentiated hDPSCs (***P < 0.001; **P < 0.01 vs hDPSCs undiff). HUVEC cells do not express PDGFR-β confirming their endothelial phenotype (Fig. 3C; Additional File 1: Fig. S1A). This result is corroborated by immunofluorescence images reported in Fig. 3D.

**Fig. 4:**
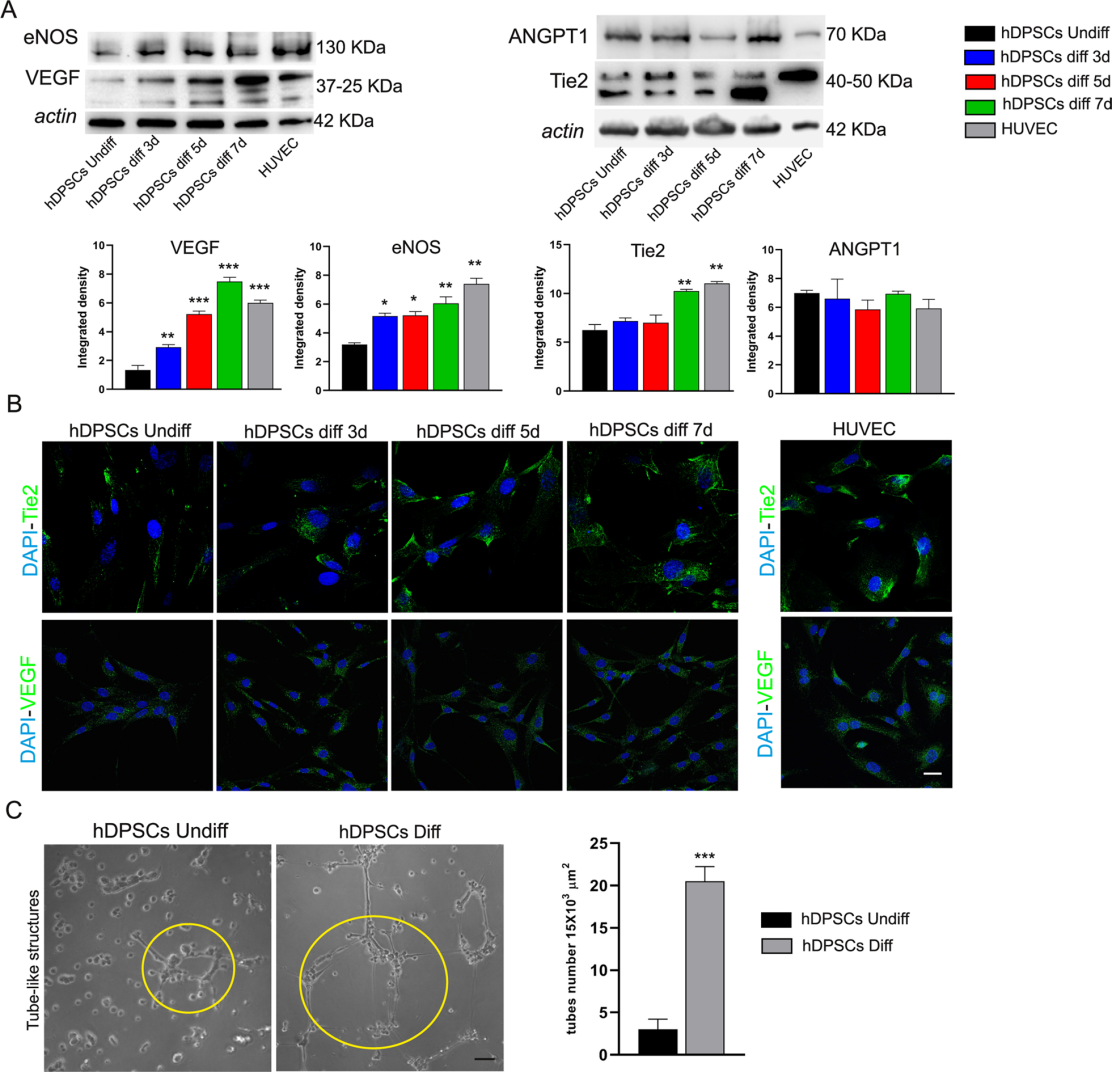
Evaluation of angiogenic potential of hDPSCs.** A** Western blot analysis of eNOS, VEGF, ANGPT1 and Tie2 in endothelial differentiated hDPSCs. hDPSCs undiff and HUVEC were used as controls. Full-length blots are reported in Additional file 1: Figure S1B. **B** The expression of Tie2 and VEGF is shown by immunofluorescence images. Nuclei were counterstained with DAPI. Scale bar: 10 μm. **C** Tube formation assay showing tube-like structures in undifferentiated and endothelial differentiated hDPSCs. Histograms reporting the mean $\pm$ SD number of tube-like structures in 15 × 10^3^ μm^2^. Scale bar: 50 μm. Data are represented as mean ± SD, and statistical analysis on A was performed by one-way ANOVA followed by Dunnett post hoc test; ***P < 0.001, **P < 0.01, *P < 0.05 vs hDPSCs undiff (*n* = 3). Statistical analysis on C was carried out by unpaired Student’s t test; ***P < 0.001 vs hDPSCs undiff (*n* = 3)

Western blot analysis of cleaved caspase 3 showed that the endothelial induction did not affect cell viability of hDPSCs (***P < 0.001 vs hDPSCs; Fig. 3E; Additional File 1: Fig. S1A). The expression of typical endothelial markers ANGPT1, Tie2, eNOS and VEGF was also investigated. Interestingly, WB analysis revealed a statistically significant upregulation of eNOS and VEGF along the endothelial differentiation time, reaching similar expressions in HUVEC that were used as positive control (***P < 0.001, **P < 0.01, *P < 0.05 vs hDPSCs undiff). Regarding the expression of Tie2 receptor, a statistically significant increase was observed only at 7 days of induction (**P < 0.01 vs hDPSCs undiff), while no statistically significant differences were observed in its ligand, ANGPT1 (Fig. 4A; Additional File 1: Fig. S1B). These data were confirmed by immunofluorescence analysis reported in Fig. 4B. Remarkably, basal levels of all these endothelial markers were expressed in undifferentiated hDPSCs suggesting their tendency to angiogenic potential (Fig. 4A and B). As a matter of fact, a functional assay was performed in order to evaluate the ability of hDPSCs to form tube-like structures. As highlighted in Fig. 4C, the presence of tube-like vascular network formation was also observed in undifferentiated hDPSCs (3 ± 1.2 tubes in 15 × 10^3^ μm^2^). This ability was strongly increased in hDPSCs upon the induction to endothelial differentiation (20.5 ± 1.73 tubes in 15 × 10^3^ μm^2^, ***P < 0.001vs hDPSCs undiff; Fig. 4C).

### Analysis of the effects mediated by flow-dependent shear stress

**Fig. 5:**
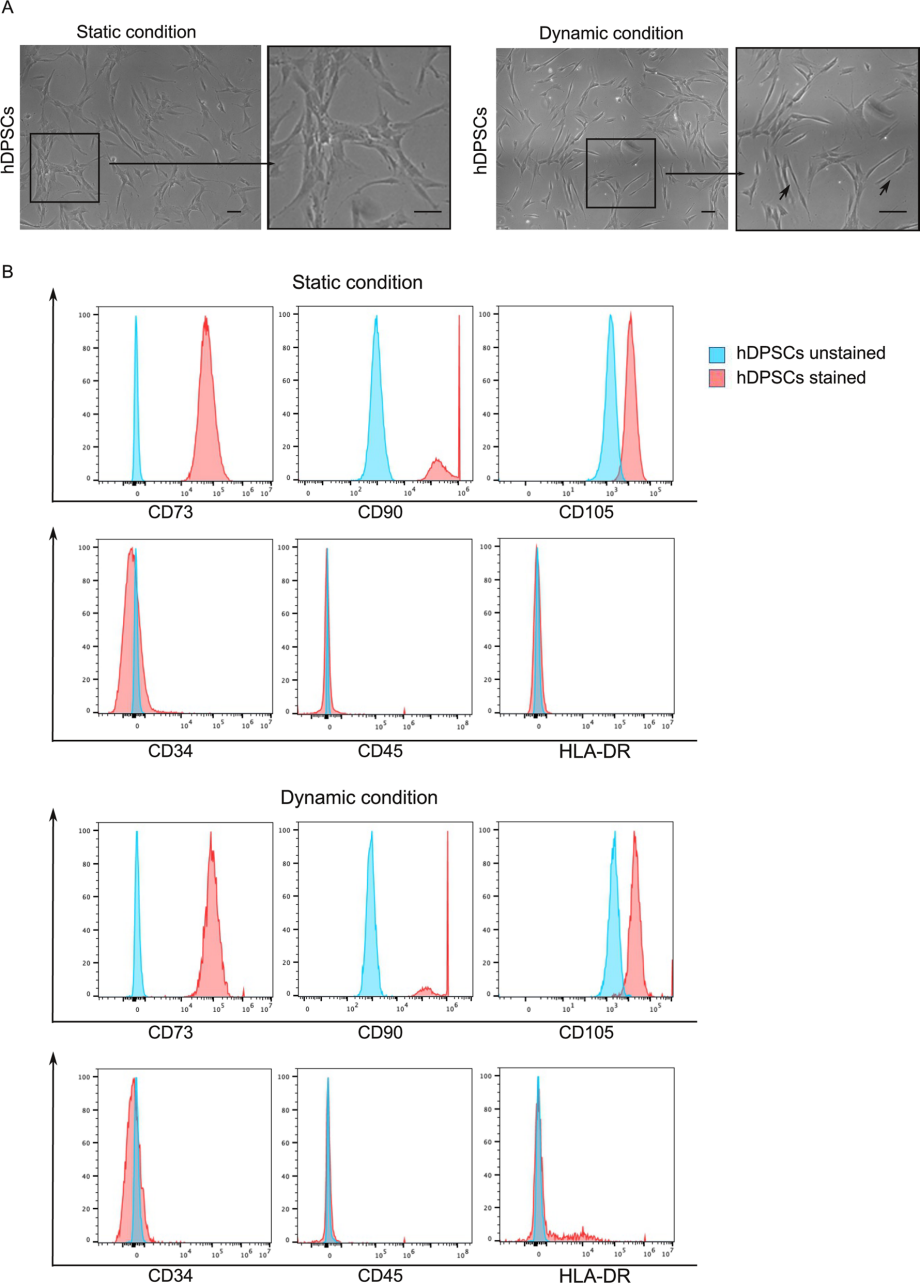
Unidirectional pulsatile flow shear stress effects on hDPSCs. **A** Evaluation of hDPSCs morphology after culture under static and dynamic conditions. Black squares and arrows indicate higher magnification and cell morphology, respectively. Scale bar: 50 μm. **B** FACS analysis of MSCs typical markers (CD73, CD90, CD105, CD34, CD45, HLA-DR) on hDPSCs after static and dynamic culture conditions

Blood vessels are regularly exposed to various types of hemodynamic forces (flow-dependent shear stress, FSS) induced by pulsatile blood flow and pressure. Shear stress can play a critical role in vascular homeostasis and remodeling as well as in the pathophysiology process by acting on endothelial cells and/or pericytes [28]. Many cellular structures, including cell membrane that represents the first layer exposed to external stimuli, respond to shear stress by adapting their shape. Based on this consideration, we noticed that hDPSCs exposed to FSS rearranged their morphology (Fig. 5A). Cells consequently appeared more elongated, losing their typical fibroblast-like morphology (Fig. 5A, black arrows). In order to exclude the possibility that FSS induced an alteration in stemness phenotype, FACS analysis was carried out on hDPSCs cultured in standard static conditions and on hDPSCs exposed to FSS, i.e., dynamic conditions. Our results showed that the FSS did not alter the expression of typical MSCs markers on hDPSCs from both experimental groups. Indeed, as reported in Fig. 5B almost all hDPSCs were positive for CD73, CD90 and CD105 while being negative for CD34, CD45 and HLA-DR (Fig. 5B).

**Fig. 6:**
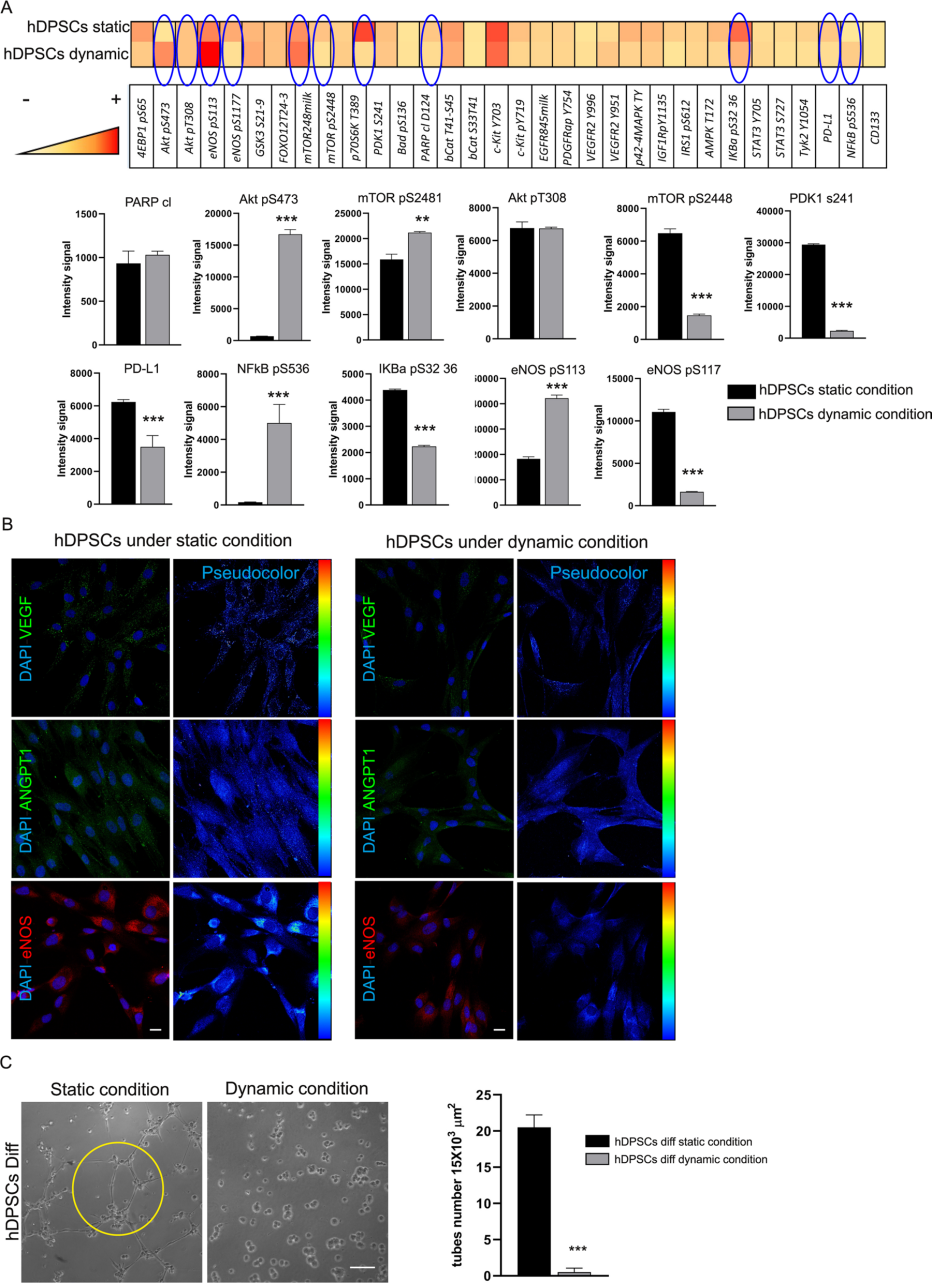
Mechanotransduction induced in hDPSCs by FSS.** A** Heatmap of RPPA analysis carried out on phosphorylated proteins in hDPSCs, after static and dynamic conditions. Histograms showing the most prominent signaling pathways. Data are represented as mean ± SD, and statistical analysis was carried out by unpaired Student’s t test; ***P < 0.001, **P < 0.01 vs hDPSCs in static condition (*n* = 5). **B** Immunofluorescence analysis carried out on hDPSCs, under static and dynamic conditions, shows the expression of endothelial markers VEGF, ANGPT1 and eNOS. Pseudocolor analysis of each endothelial marker is reported on the right side. Nuclei were counterstained by DAPI. Scale bar: 10 μm. **C** Tube formation assay showing tube-like structures in endothelial differentiated hDPSCs under static and dynamic conditions. Histograms reporting the mean $\pm$ SD number of tube-like structures in 15 × 10^3^ μm^2^. Scale bar: 50 μm. Data are represented as mean ± SD and statistical analysis was carried out by unpaired Student’s *t* test; ***P < 0.001 vs hDPSCs diff static condition (*n* = 5)

Following the initial mechanosensing reflected on hDPSCs membrane modifications, the activation of local biochemical responses and downstream intracellular signaling pathways might have occurred [29]. In particular, we evaluated different activated pathways on hDPSCs exposed to FSS. As shown in Fig. 6A, heatmap and histograms revealed the activation of different signaling mechanisms. First of all the FSS did not induce pro-apoptotic signaling in hDPSCs as revealed by the lack of variation in the expression of cleaved PARP protein in both experimental groups. The major finding of the present analysis is that Akt/mTOR pathway was activated in response to pulsatile unidirectional flow-dependent shear stress. As shown, the statistical up-regulation of Akt pS473 was correlated with the statistically significant increase in mTOR pS2481 in hDPSCs under dynamic conditions when compared to hDPSCs under static conditions (***P < 0.001, **P < 0.01 dynamic condition vs static condition). In parallel, the phosphorylation of Akt pT308 did not change in both experimental conditions and the phosphorylation of PDK1 s241 decreased in statistically significant manner in hDPSCs exposed to FSS (***P < 0.001 vs static condition). These data indicate the Akt/mTOR pathway activation is due to hDPSCs membrane deformation and mechanosensing activation. Moreover, remarkably the FSS induced a statistically significant decrease in PD-L1 immunomodulatory marker. In parallel, hDPSCs maintained in dynamic conditions displayed a strong up-regulation of NFkB pS536, along with a statistically significant decrease in IKBα pS32 36, whose phosphorylation is reported to be linked with NFkB inactivation (***P < 0.001 vs static condition). These data could suggest that pulsatile flow shear stress regulates the pro-inflammatory fate of hDPSCs. Interestingly, we noticed that the FSS induced a statistically significant downregulation of eNOS pS117 (***P < 0,001 vs static condition) and in parallel a statistical significant up-regulation of eNOS pS113 (***P < 0,001 vs static condition). These data suggest that eNOS activity was inhibited as well as FSS affect angiogenic potential tendency of hDPSCs. This data was confirmed by immunofluorescence staining of typical angiogenic markers (VEGF, ANGPT1 and eNOS) and by tube formation assay. In particular as revealed by pseudocolor analysis, the expression of VEGF, ANGPT1 and eNOS decreased in hDPSCs exposed to dynamic culture conditions (Fig. 6B). At the same time, the functional analysis also revealed a statistically significant reduction in the count of tube-like structures in hDPSCs under dynamic condition (0.5 ± 0.5 tubes in 15 × 10^3^ μm^2^; ***P < 0.001) when compared with the hDPSCs maintained in static culture condition (21 ± 1.5 tubes in 15 × 10^3^ μm^2^; Fig. 6C), thus confirming that the FSS also influenced the ability of hDPSCs to form tube-like vascular networks and subsequently their angiogenic potential.

### Analysis of the immunomodulatory and inflammatory response of hDPSCs to flow-dependent shear stress

**Fig. 7:**
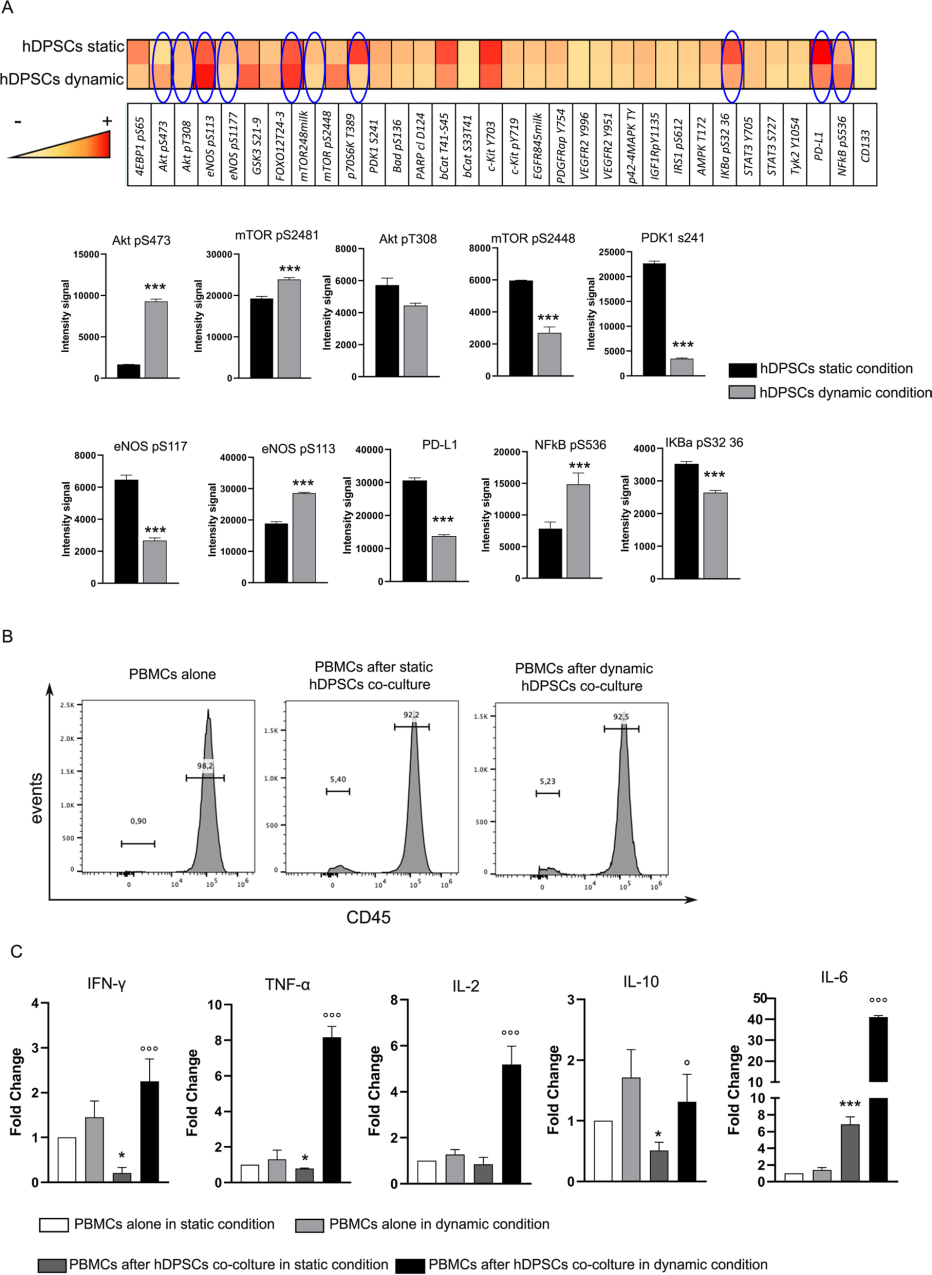
Effects of inflammatory microenvironment on hDPSCs exposed to FSS. **A** Heatmap of RPPA analysis carried out on phosphorylated proteins in hDPSCs, after co-culture with PBMCs in static and dynamic conditions. Histograms showing the most prominent signaling pathways. Data are represented as mean ± SD, and statistical analysis was carried out by unpaired Student’s *t* test; ***P < 0.001, **P < 0.01 vs hDPSCs in static condition (*n* = 5). **B** FACS analysis of CD45 on PBMCs following static and dynamic co-culture with hDPSCs. **C** Real-time PCR analysis of different cytokines was performed on PBMCs after hDPSCs co-culture in static and dynamic conditions. Histograms represent the mean ± SD mRNA fold-change (*n* = 5). Statistical analysis was carried out by one-way ANOVA followed by Newman–Keuls post hoc test. ***P < 0.001, *P < 0.05 vs. PBMCs alone in static condition; °°°*P* < 0.001, °P < 0.05 vs PBMCs after DPSCs co-culture in static conditions

The effect of dynamic flow conditions on the inflammatory microenvironment mimicked by the co-culture between hDPSCs and pre-activated CD3/CD28 hPBMCs was investigated (Fig. 7A). The biological analysis was carried out by RPPA on hDPSCs after co-culture with PBMCs with or without FSS. As demonstrated above, we first confirmed that mechanosensing transduction activated the pathway of Akt/mTOR. Particularly, as reported in Fig. 7A, histograms showed a statistically significant increase in Akt pS473 that correlated with a statistically significant increase in mTOR pS2481 in hDPSCs under dynamic conditions, when compared to hDPSCs under static conditions (***P < 0.001 hDPSCs dynamic condition vs hDPSCs static condition). Moreover, no statistically significant difference was detected in the phosphorylation of Akt pT308 in both experimental conditions, whereas the phosphorylation of PDK1 S241 statistically significant decreased in hDPSCs after co-culture with PBMCs exposed to FSS (***P < 0.001 vs static condition). In this co-culture system, the eNOS activity of hDPSCs exposed to FSS was inhibited, as demonstrated by the significant downregulation of eNOS pS117 (***P < 0.001 vs hDPSCs static condition) and, at the same time, by the statistically significant upregulation of eNOS pS113 (***P < 0.001 vs hDPSCs static condition, Fig. 7A). Subsequently, we investigated the immunomodulatory properties in hDPSCs exposed or not to FSS after co-culture with PBMCs. Interestingly, the upregulation of PD-L1 was confirmed in hDPSCs in static condition in accordance with our previous findings. The application of FSS drastically decreased (more than 60%) the expression of PD-L1 in hDPSCs in dynamic condition when compared to hDPSCs in static condition (***P < 0.001 vs static condition; Fig. 7A). At the same time, an evident increase in NFkB pS536, as well as a significant reduction of IKBα pS32 36, was revealed in hDPSCs under dynamic condition after co-culture with PBMCs (***P < 0.001 vs static condition; Fig. 7A), suggesting that a pro-inflammatory fate is driven by FSS.

In order to confirm the plasticity of hDPSCs in immunomodulation, we evaluated the intracellular inflammatory cytokines in pre-activated PBMCs after co-culture with hDPSCs in static and dynamic condition. To this regard, FACS analysis showed a minimal and comparable contamination of hDPSCs in PBMCs after either static or dynamic co-culture (Fig. 7B). In detail, the FSS was able to induce an up-regulation trend of mRNA expression levels of all cytokines tested in pre-activated PBMCs alone in dynamic conditions when compared to the counterpart in static conditions. Furthermore, histograms showed a strong significant increase in mRNA expression levels of IFNγ, TNFα, IL-2, IL-10 and IL-6 in PBMCs after hDPSCs co-culture in dynamic condition (°°°*P* < 0.001, °*P* < 0.05 vs PBMCs after hDPSCs co-culture in static condition, Fig. 7C). Conversely, a downregulation of cytokines levels (IFNγ, TNFα, IL-2, IL-10) was detected in PBMCs after hDPSCs co-culture in static condition, when compared with PBMCs cultured alone in static condition (*P < 0.05; Fig. 7C), except for IL-6 that was up-regulated (***P < 0.001) after hDPSCs co-culture in static condition, which is in accordance with our previous findings (Fig. 7C) [7, 12]. Taken together these data suggest that FSS exerted a strong effect on pro-inflammatory behavior of hDPSCs.

## Discussion

Pericytes are mural cells that surround the endothelium of small vessels. They regulate numerous functions including vessel growth, stabilization and permeability [30]. Defining a pericyte is a challenge since no unique molecular marker has been identified because of the different embryological origin, functions and locations in various tissues. A large number of studies have suggested that pericytes behave as MSCs. Indeed, besides the expression of PDGFRβ, pericytes express typical MSCs markers, are able to adhere to tissue culture plastic, can be expanded in vitro for multiple passages and can differentiate into different cell lineages. [27, 31]. Pericytes have been strongly associated with vessel formation and stabilization as well as hemodynamic processes of blood vessels, and it is suggested that either direct and paracrine interplay between pericytes and endothelial cells exists [3, 32–36]. It is well known that pericytes have an angiogenic and vasculogenic potential in enhancing blood vessel formation [37].

In our study, we have demonstrated that STRO-1 + /c-Kit + human dental pulp stem cells are able to express both typical MSCs markers and PDGFRβ, suggesting their pericyte-like phenotype, in accordance with previous findings [13, 38].

In the first phase of our study, we have observed that hDPSCs are able to express at basal levels, in undifferentiated status, the typical endothelial markers, i.e., Tie2, eNOS, ANGPT1 and VEGF that are upregulated after culture in the endothelial induction medium, thus confirming that hDPSCs are naturally prone toward endothelial differentiation. These findings are supported by the ability of hDPSCs to form tube-like structures as early as in undifferentiated status. Notably, angiogenic signaling including ANGPT1 and Tie2 was detected in hDPSCs in standard conditions and maintained also after endothelial induction. To this regard, it is well known that ANGPT1/Tie2 signaling is able to regulate two opposite processes, i.e., vascular quiescence and angiogenesis [39], and has also been proven to exert potent anti-inflammatory effects, by inhibiting NF-kB, a transcription factor involved in inflammatory processes [40].

Interestingly, the ANGPT1/Tie2 signaling might be influenced by mechanical forces including pulsatile shear stress, suggesting that FSS plays a pivotal role in modulating the endothelial phenotype of pericyte-like cells and, likely, their immunomodulatory/inflammatory behavior as well [28]. Our data demonstrate that when a pulsatile unidirectional flow is applied to hDPSCs their stemness phenotype was not altered, however a shift in cell morphology was observed. As a matter of fact, it is well known that fluid shear stress induces deformation of cell membranes. This event, called mechanosensing, consists in the transformation of mechanical stress into biochemical signals, inducing the activation of downstream intracellular signaling pathways, alterations of gene and protein expression, which result in modification of cell function [29].

In light of our results, the endothelial differentiation potential of hDPSCs exposed to FSS was inhibited as shown by the downregulation of pro-angiogenic phosphorylation of eNOS S1177 [31] and the simultaneous upregulation of anti-angiogenic phosphorylation of eNOS S113 [42]. Moreover, we observed a decreased expression of VEGF and ANGPT1 as well as the loss of tube formation ability. Our findings highlighted that these cell functional alterations are strictly related to mechanically dependent activation of biochemical signaling pathways, as demonstrated either by the increased expression of Akt pS473/mTOR pS2481 and the lack of activation in Akt pT308/mTOR pS2448, in accordance with findings from Dimmeler et al. [43]

It is noteworthy that the pulsatile unidirectional flow application induced the downregulation of the immune-modulatory molecule PD-L1. As demonstrated by our recent studies, hDPSCs are able to modulate the inflammatory microenvironment by exerting immunomodulatory properties through the upregulation of PD-L1 [8].

This downregulation might reflect hDPSCs plasticity in exerting immunomodulation, switching their potential from an anti-inflammatory toward a pro-inflammatory fate. The latter one might indeed be linked to the observed upregulation of NFkB pS536 [44].

To this purpose, we aimed to further demonstrate our hypothesis by co-culturing hDPSCs in an inflammatory microenvironment in the presence of FSS. Evidence from these culture conditions confirmed the data observed above and the upregulation of PD-L1 was observed in hDPSCs maintained in static co-culture conditions, thus confirming our previous findings [7]. Interestingly, when FSS was applied to hDPSCs/PBMCs co-culture, a significant reduction of PD-L1 was detected, together with a stronger upregulation of NFkB pS536. These data strengthen the ability of flow-dependent shear stress to modulate the immunomodulatory features of hDPSCs that, when also exposed to an inflammatory microenvironment mimicked by pre-activated PBMCs, further drop their potential to express immunomodulatory molecules and, at the same time, move toward a pro-inflammatory behavior.

Additional proof of this event resulted from the significantly up-regulated expression levels of all the evaluated pro-inflammatory cytokines expressed by PBMCs. These findings are definitely countertrend when compared to data obtained from co-culture experiments in static conditions, which are still consistent with evidence from our recent studies [7, 12].

## Conclusions

Taken together all these observations, it might be argued that mechanical forces may determine the biological fate of hDPSCs, which might be activated during the development of diseases driven by the establishment of an inflammatory microenvironment, such as autoimmune diseases and precancerous lesions. To this regard, it is known that MSCs hold an active interplay with immune cells and consequently may display both anti-inflammatory and pro-inflammatory effects by acting as sensors and switchers of inflammation [45]. Moreover, these data pave the way to understand the potential role of pericyte-like cells and mechanical forces in the trigger and development of inflammation-dependent diseases.

## Supplementary Information

**Additional file 1: Figure S1.** Extended data for figure 3 and 4. Uncropped Western blot images showing **A** PDGFR-β and related actin; **B** cleaved caspase-3 and related actin; **C** eNOS and VEGF with related actin; **D** Tie2, ANGPT1 and related actin. Boxed areas correspond to cropped regions shown in figure 3C and 3E. Specific target bands were selected according to the molecular weight reported in antibodies’ datasheets. **Table S1**: raw RPPA data.

## Data Availability

All data generated or analyzed during this study are included in this article.
