# Supplementary material for: Flow-dependent shear stress affects the biological properties of pericyte-like cells isolated from human dental pulp
Source: Stem Cell Res Ther. 2023 Feb 18;14:31. doi: 10.1186/s13287-023-03254-2 (PMC9938980; doi:10.1186/s13287-023-03254-2)

Additional File 1

Flow-dependent shear stress affects the biological properties of pericyte-like cells isolated

from human dental pulp

1

*

1*

1*

1

1

Giulia Bertani , Rosanna Di Tinco , Laura Bertoni , Giulia Orlandi , Alessandra Pisciotta ,

2

3

4

1

1

Roberto Rosa , Luca Rigamonti , Michele Signore , Jessika Bertacchini , Paola Sena , Sara De

Biasi^5^, Erica Villa_1✝_, Gianluca Carnevale_1✝°_

^1^Department of Surgery, Medicine Dentistry and Morphological Sciences with Interest in

Transplant, University of Modena and Reggio Emilia, Modena, Italy

^2^Department of Engineering Sciences and Methods, University of Modena and Reggio Emilia,

Modena, Italy

^3^Department of Chemical and Geological Sciences, University of Modena and Reggio Emilia,

Modena, Italy

^4^RPPA Unit, Proteomics Area, Core Facilities, Istituto Superiore di Sanità, Rome, Italy

^5^Department of Medical and Surgical Sciences for Children and Adults, University of Modena and

Reggio Emilia, Modena, Italy.

Supplementary figure 1. Extended data for figure 3 and 4. Uncropped Western blot images

showing A PDGFR-β and related actin; B) cleaved caspase-3 and related actin; C) eNOS and

VEGF with related actin; D) Tie2, ANGPT1 and related actin. Boxed areas correspond to cropped

regions shown in figure 3C and 3E. Specific target bands were selected according to the molecular

weight reported in antibodies’ datasheets.


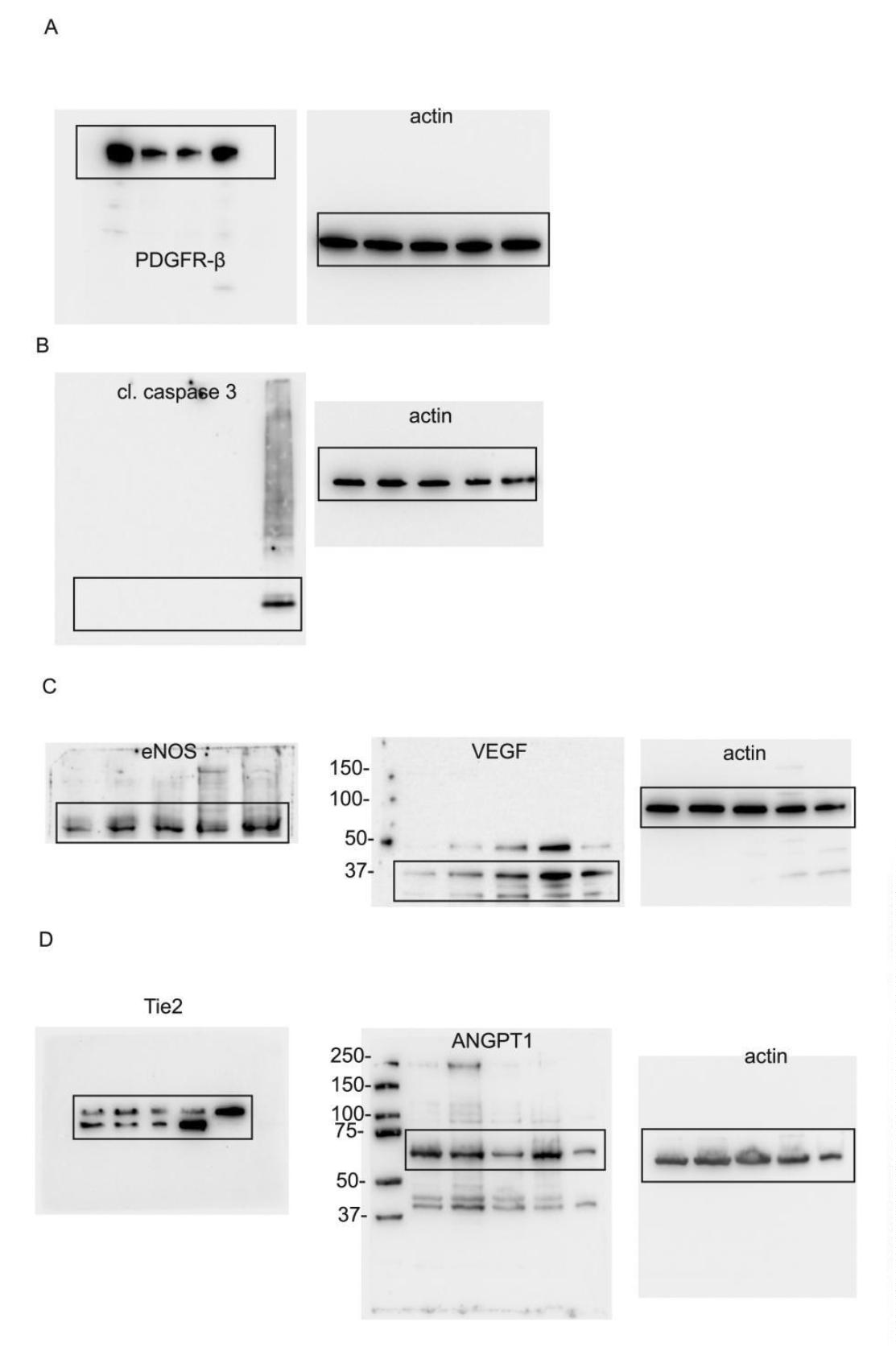


**Supplementary Table 1: raw RPPA data**

**Antibodies**

**hDPSCs alone static hDPSCs alone dynamic**

3440

12913

3989

93

683

03

762

6400

144

8270

17448

**hDPSCs co-culture static hDPSCs co -culture dynamic**

1

5410

5367

5453

16714

15994

17466

6741

6661

6823

42193

40946

43478

1628

1573

1684

11070

8920

13739

7023

6516

7570

21184

20973

21397

1470

1391

1553

5156

4846

5486

13121

12345

13947

1657

1633

1682

5710

5287

6167

18902

18343

19477

6464

6173

6768

8928

8743

9118

4741

4329

5193

19264

18732

19811

5961

5931

5991

5814

5620

6015

4727

4196

*4*

*EBP1 pS65*

1

5324

6

9284

*Akt pS473*

9000

7

9576

6

4438

*Akt pT308*

4286

7

4596

1

28567

28283

28854

2665

*eNOS pS113_L*

*eNOS pS1177*

*GSK3 S21-9_L*

*FOXO12T24-3_L*

*mTOR248milk_L*

*mTOR pS2448*

*p70S6K T389*

1

1

9130

1059

10743

1384

109

8964

2505

1

2836

9

18034

17052

19072

8973

9

7

256

495

7325

669

5867

14839

6967

490

6235

7887

7

10209

23861

23389

24343

2678

1

1

6

2331

6

4

755

390

3078

14574

14228

14928

4004

4813


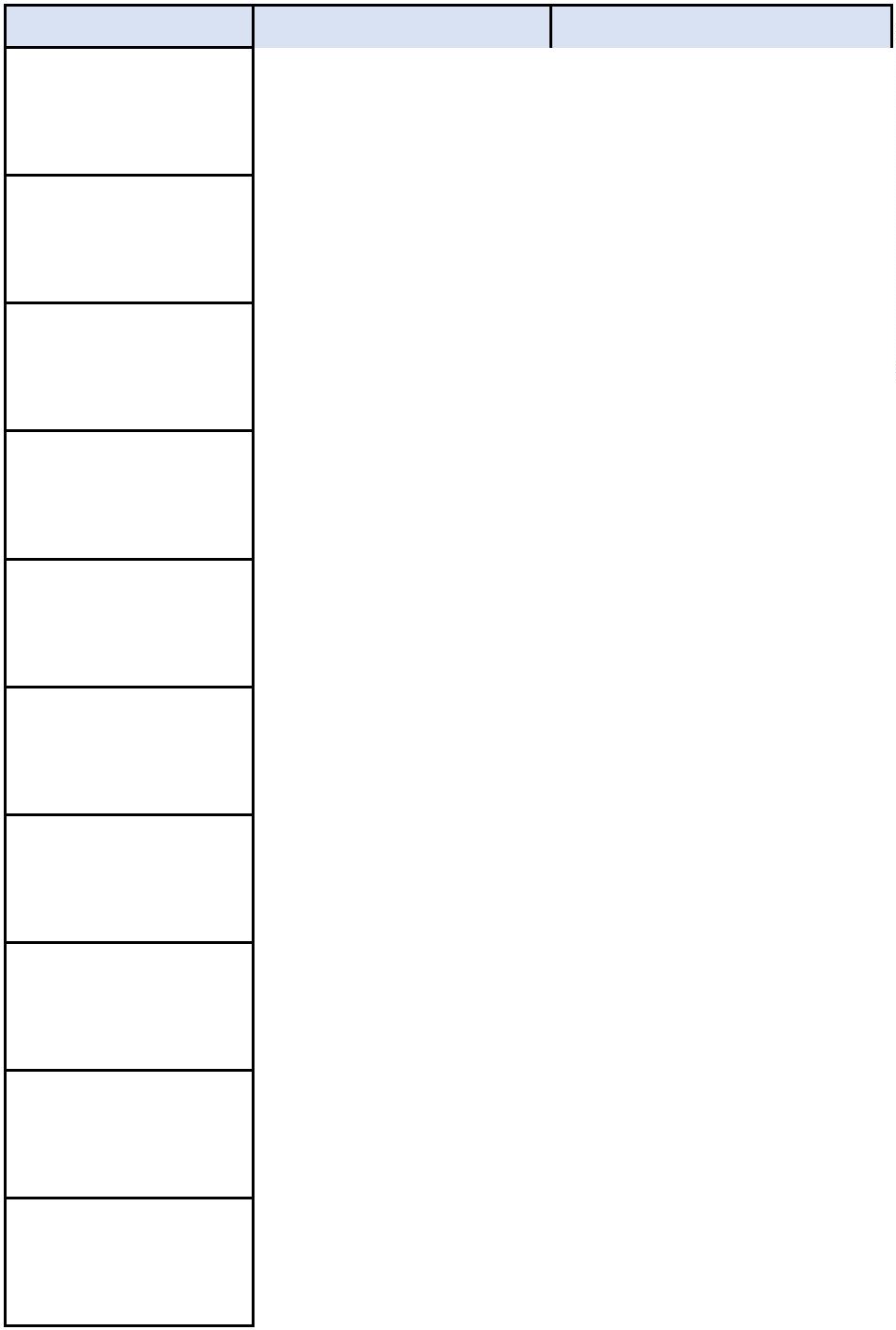

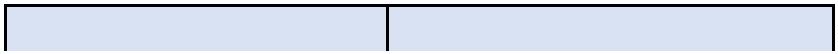


2

9437

29144

9733

115

6995

237

93

679

27

198

6045

355

3684

12481

2271

2082

2477

6124

5825

6438

1063

983

22697

22248

23156

8039

7824

8259

6108

5591

6635

4813

4680

4949

20848

20435

21269

899

3484

3351

3623

5659

5255

6094

3194

3124

3265

2458

2340

2581

12040

11920

12161

401

*PDK1 S241*

*Bad pS136_L*

*PARP cl D124*

*bCat T41-S45*

*bCat S33T41_L*

*c-Kit Y703*

2

7

7

7

9

1149

2380

2180

2599

10668

10572

10764

402

6

6

1

1

5003

70

751

88

8283

7

346

883

383

7

466

915

420

2

23156

22471

23861

3678

3314

4081

2338

833

25084

23861

26370

5351

5110

5603

4876

4661

5100

6355

5931

6809

1045

874

18996

18883

19111

3711

3594

3831

2308

1321

4032

3835

3565

4126

1152

1115

1189

*c-Kit pY719*

28001

8567

967

6380

2

6

*EGFR845milk_L*

*PDGFRap Y754_L*

*VEGFR2 Y996*

*VEGFR2 Y951_L*

7

4

608

064

3862

4

3

277

801

6562

3512

3344

3689

1581

1442

1734

3641

968

26

527

98

3

7

9

1250


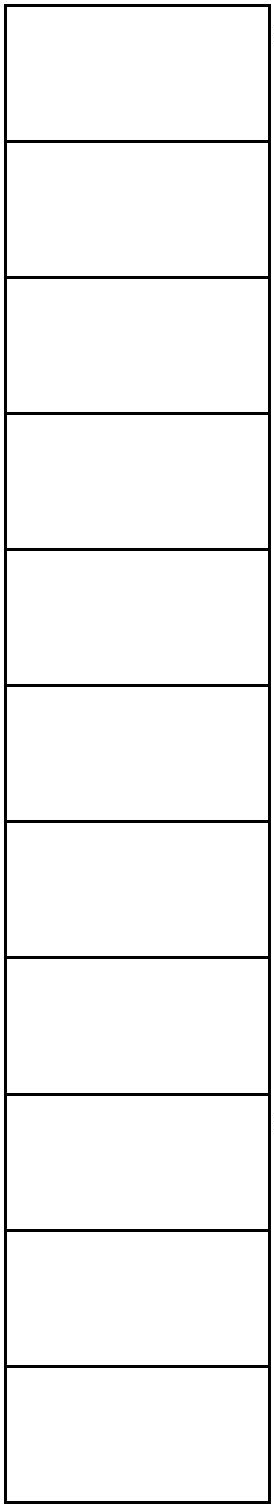


2

298

5997

5908

6088

3843

2984

4949

5182

4661

5762

820

2445

2141

2793

4346

4167

4532

6015

5890

6143

2870

2592

3178

3523

3450

3598

4876

4569

5203

3583

3530

3637

3519

3474

3565

31257

30857

29728

7809

6836

8920

67.4

2071

1908

2248

2399

1916

3005

5029

4789

5282

2487

2350

2633

2644

2579

2711

4760

3562

6361

1556

1441

1681

1815

1765

1867

13767

13373

14172

14794

13108

16697

83.6

*p42-4MAPK TY*

*IGF1RpY1135_L*

*IRS1 pS612_L*

*AMPK T172*

*IKBa pS32 36*

*STAT3 Y705_L*

*STAT3 S727*

*Tyk2 Y1054*

*PD-L1*

2213

2

9

387

377

9293

9

8

462

283

8160

8

4

409

460

4363

786

4

4

560

390

855

2233

2191

2276

2847

2364

3429

1434

1283

1602

1594

1439

1765

2165

2043

2294

4920

3913

6186

121

4355

4

2

425

231

2088

2

2

383

553

2373

2

3

746

980

3956

4

6

004

242

6094

393

55

170

90

6.1

68.4

4.6

6

1

*NFkB pS536_L*

*CD133*

1

7

76.2

193

62.1

57.1

8

73

122


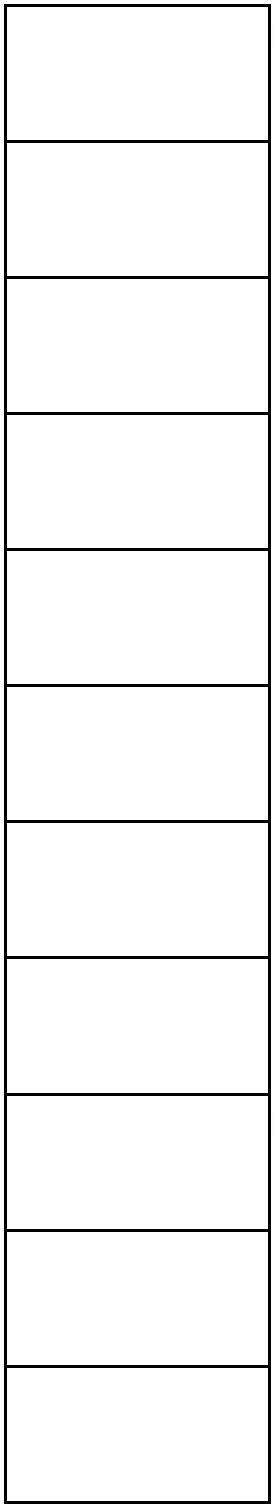

Supplement: Supplementary file 1 — Additional file 1: Figure S1. Extended data for figure 3 and 4. Uncropped Western blot images showing A PDGFR-β and related actin; B cleaved caspase-3 and related actin; C eNOS and VEGF with related actin; D Tie2, ANGPT1 and related actin. Boxed areas correspond to cropped regions shown in figure 3C and 3E. Specific target bands were selected according to the molecular weight reported in antibodies’ datasheets. Table S1: raw RPPA data. [file 13287_2023_3254_MOESM1_ESM.docx]
